# Curative Surgery for Malignant Sigmoid–Duodenal Fistula Caused by Colon Cancer: A Case Report

**DOI:** 10.70352/scrj.cr.26-0218

**Published:** 2026-06-17

**Authors:** Saho Aso, Moe Tasaki, Kenta Aso, Yuzuru Ito, Shunsuke Shirakawa, Kentaro Kawamitsu, Takuji Noro, Michio Machida, Hiroyuki Sugo

**Affiliations:** 1 Department of General Surgery, Juntendo University Nerima Hospital, Tokyo, Japan; 2 Department of Hepatobiliary Pancreatic Surgery, Juntendo University School of Medicine, Tokyo, Japan; 3 Kawamitsukeikou-kai Medical Corporation, Kawamitsu Surgery, Tokyo, Japan

**Keywords:** sigmoid–duodenal fistula, segmental duodenectomy, colon cancer, case report

## Abstract

**INTRODUCTION:**

A malignant sigmoid colon–duodenal fistula is extremely rare, and optimal management strategies remain unclear.

**CASE PRESENTATION:**

A 66-year-old man was admitted to a local hospital with melena. Lower gastrointestinal endoscopy identified a circumferential type 3 tumor located in the sigmoid colon. Upper gastrointestinal endoscopy revealed extrinsic tumor invasion into the horizontal portion of the duodenum. Contrast-enhanced abdominal CT demonstrated irregular thickening of the sigmoid colon wall, which appeared to be contiguous with the horizontal portion of the duodenum, with evidence of luminal continuity. Based on a diagnosis of malignant sigmoid–duodenal fistula, the patient underwent open surgery. At laparotomy, the sigmoid colon cancer was found to be directly invading the duodenojejunal flexure, arising from the ligament of Treitz on the left side of the superior mesenteric artery, which made preservation of the duodenum difficult. Therefore, Hartmann’s procedure with concomitant segmental duodenectomy was performed while preserving the pancreas. For duodenal reconstruction, a Roux-en-Y jejunal limb was created and anastomosed to the second portion of the duodenum. Additionally, a gastrojejunostomy in the Billroth II configuration was performed.

**CONCLUSIONS:**

Malignant invasion of the duodenum is often associated with involvement of major vessels or the need for pancreatic resection, which may render curative resection difficult or excessively invasive. However, segmental duodenectomy with preservation of the pancreas can be a valuable surgical option in selected cases.

## Abbreviations

DGE
: delayed gastric emptying
PD
: pancreatoduodenectomy
SMA
: superior mesenteric artery
SMA/V
: superior mesenteric artery and vein

## INTRODUCTION

Duodenocolic fistula secondary to colon cancer is rare, with a reported incidence of approximately 0.1% of all colon cancer cases.^1)^ Although there have been reports of fistula formation caused by duodenal invasion of colorectal cancer, most of them affected the transverse colon, which is anatomically adjacent to the duodenum.^2)^ On the other hand, a sigmoid colon–duodenal fistula is extremely rare. Consequently, optimal management strategies and the most appropriate surgical procedures for this condition remain unclear.

Herein, we report a rare case of a sigmoid colon–duodenal fistula caused by direct invasion of sigmoid colon cancer, which was successfully diagnosed preoperatively and curatively resected.

## CASE PRESENTATION

A 66-year-old man was admitted to a local hospital 2 weeks prior with melena and abdominal distension. Other than this, he had no significant medical history. During hospitalization, he was managed with bowel rest (*nil per os*). On the basis of diagnostic evaluations, colorectal cancer was suspected, and he was referred to Juntendo University Nerima Hospital for surgical management. On admission, abdominal radiography revealed marked colonic gas accumulation and colonic dilatation (**Fig. 1**). Laboratory tests revealed severe anemia and malnutrition: hemoglobin 6.9 g/dL (normal range: 13.0–17.0 g/dL), C-reactive protein 5.1 mg/dL (normal range: 0.0–0.3 mg/dL), total protein 5.5 g/dL (normal range: 6.4–8.3 g/dL), and albumin 1.9 g/dL (normal range 3.8–5.2 g/dL). Tumor markers, including carcinoembryonic antigen (1.1 ng/mL; normal range 0.0–0.5 ng/mL) and carbohydrate antigen 19-9 (5.1 U/mL; normal 0–37 U/mL), were within normal limits. Lower gastrointestinal endoscopy identified a circumferential type 3 tumor located 20 cm from the anal verge (**Fig. 2A**). Further advancement of the endoscope beyond the lesion was difficult because of severe stenosis, and biopsy specimens revealed adenocarcinoma. Upper gastrointestinal endoscopy revealed extrinsic tumor invasion into the horizontal portion of the duodenum, suspected to originate from the sigmoid colon cancer, and biopsy specimens confirmed adenocarcinoma (**Fig. 2B**). Contrast-enhanced abdominal CT demonstrated irregular thickening of the sigmoid colon wall, which appeared to be contiguous with the horizontal portion of the duodenum and showed evidence of luminal continuity (**Fig. 3**). These findings suggested duodenal invasion by sigmoid colon cancer. No distant metastases were detected, and the patient subsequently underwent surgery based on a diagnosis of sigmoid colon cancer with a malignant sigmoid–duodenal fistula. An open surgical approach was selected to allow adequate assessment of duodenal invasion and operability. Preoperatively, the patient presented with malnutrition due to obstructive colorectal cancer. Therefore, Hartmann’s procedure was planned for the primary lesion in order to avoid colorectal anastomosis.

**Fig. 1:**
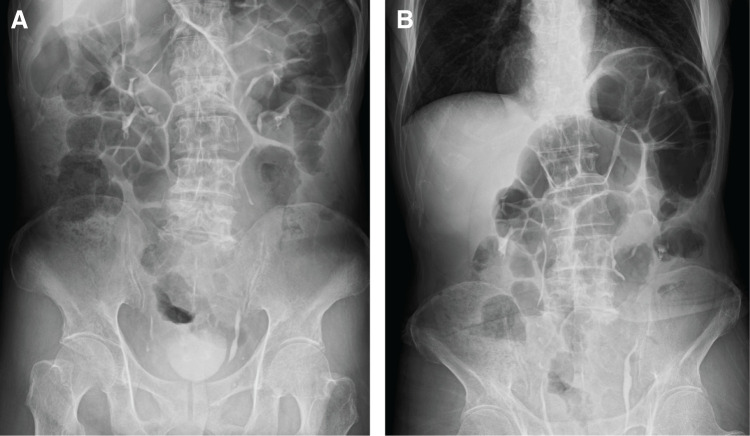
Abdominal radiograph. (**A**) Upright position; (**B**) supine position. Dilated bowel loops were observed on plain abdominal radiography.

**Fig. 2:**
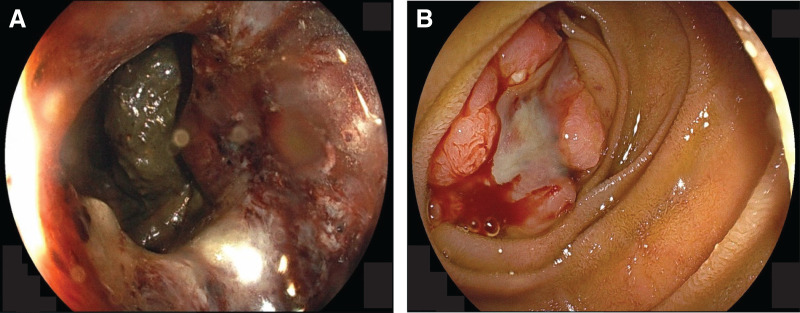
Gastrointestinal endoscopic findings. (**A**) Lower gastrointestinal endoscopy of the sigmoid colon demonstrated a type 3 tumor located 20 cm from the anal verge. (**B**) Upper gastrointestinal endoscopy revealed an irregularly shaped tumor in the third portion of the duodenum.

**Fig. 3:**
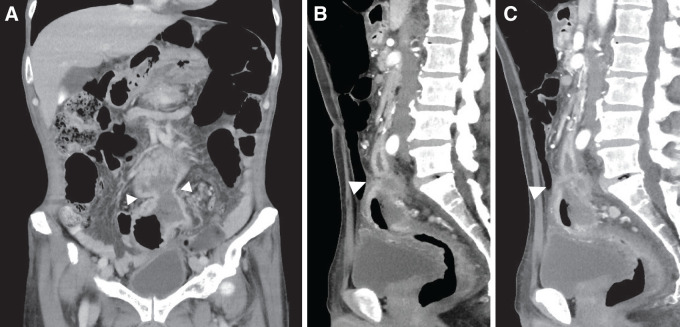
Contrast-enhanced abdominal CT imaging. (**A**) Coronal view demonstrated continuity between the lumina of the sigmoid colon and the horizontal portion of the duodenum, suggesting direct duodenal invasion by sigmoid colon cancer (arrowheads). (**B**, **C**) Sagittal views similarly revealed continuity between the lumina of the sigmoid colon and duodenum, accompanied by bowel wall thickening and enlargement of regional lymph nodes adjacent to the sigmoid colon (arrowhead).

Laparotomy revealed that the colon cancer was located in the middle portion of the sigmoid colon and had directly invaded the duodenojejunal flexure arising from the ligament of Treitz on the left side of the SMA (**Fig. 4A**). Although no direct invasion of the SMA or pancreatic parenchyma was found, more than half of the circumference of the duodenum was involved, making duodenal preservation difficult (**Fig. 4B**). Therefore, sigmoid colectomy with concomitant resection of the horizontal portion of the duodenum (segmental duodenectomy) was performed. First, Kocher mobilization was performed to free the SMA/V pedicle and the invaded area *en bloc* from the right side. Subsequently, from the left side, the SMA/V and duodenum were carefully dissected and separated, and only the invaded portion was encircled. After division of the ligament of Treitz, the horizontal portion of the duodenum was dissected while preserving the pancreas. The oral margin of the duodenal invasion was mobilized as far to the left of the SMA as possible to ensure an adequate safety margin, and transection was performed using a stapling device at a site located almost posterior to the SMA. The anal side of the duodenum was then transected in a similar manner, securing a safety margin of approximately 10 cm from the invaded region. After complete mobilization of the invaded duodenal segment, Hartmann’s procedure was performed for the sigmoid colon cancer, and the tumor, including the invaded portion of the duodenum, was resected *en bloc*.

**Fig. 4:**
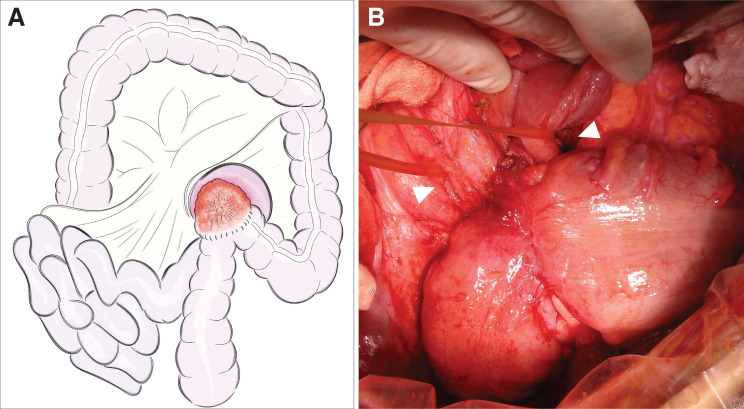
Intraoperative findings. (**A**) Schematic illustration of intraoperative findings. (**B**) Intraoperative examination revealed firm adhesion of the sigmoid colon cancer to the horizontal portion of the duodenum immediately distal to the ligament of Treitz (arrowheads). *En bloc* resection of the involved segments of the duodenum and sigmoid colon was performed.

For duodenal reconstruction, primary anastomosis at the duodenal stump was considered difficult because the stump was located dorsal to the SMA; therefore, the oral duodenal stump was closed. For drainage of the blind end of the duodenum, a Roux-en-Y jejunal limb was constructed, and a side-to-side duodenojejunostomy was performed at the second portion of the duodenum using a stapling device. The enterotomy was closed with hand-sewn sutures, with careful attention paid to avoid anastomotic stenosis. Subsequently, a gastrojejunostomy with Braun anastomosis was additionally performed 60 cm distal to the duodenojejunostomy using the same technique (**Fig. 5**).

**Fig. 5:**
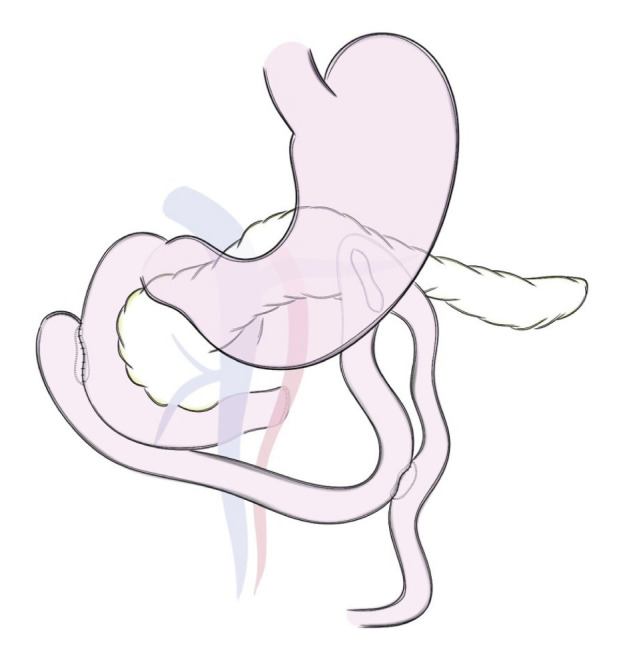
Surgical schema. Reconstruction was performed with duodenojejunostomy, gastrojejunostomy, and Braun anastomosis.

Examination of the resected specimen demonstrated a type 3 tumor measuring 60 × 60 mm in the sigmoid colon (**Fig. 6**). The predominant histology of the tumor was moderately differentiated tubular adenocarcinoma (tub2), with focal well-differentiated components. The depth of tumor invasion was classified as pT4b (i.e., beyond the duodenum). Both lymphatic invasion (ly1b) and venous invasion (v1b) were evident. Perineural invasion (Pn1b) and tumor budding (BD1) were also identified. A total of 22 regional lymph nodes were examined, but none showed metastatic involvement (pN0). No distant metastasis was identified (pM0). The proximal and distal resection margins were free of tumor involvement (pPM0 and pDM0). On the basis of the TNM classification (UICC 8th edition), the tumor was staged as pStage IIC,^3)^ and based on the Japanese Society for Cancer of the Colon and Rectum guidelines (2024 edition), the final pathological stage was pStage IIc.^4)^

**Fig. 6:**
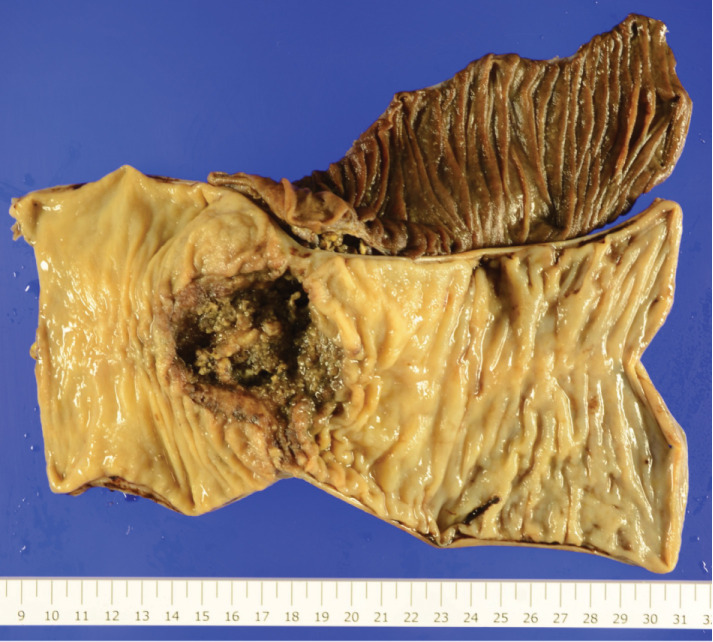
The resected specimen. Macroscopic examination of the resected specimen demonstrated a 60-mm type 3 tumor in the sigmoid colon, diagnosed as moderately differentiated tubular adenocarcinoma. The tumor showed transmural invasion of the sigmoid colon with direct extension into the duodenum, resulting in the formation of a sigmoid–duodenal fistula.

Postoperatively, the patient developed urinary dysfunction, which improved with conservative management, and on POD 36, he was transferred to another hospital for rehabilitation. Because of marked deterioration in his physical condition, postoperative adjuvant chemotherapy was not administered. At 7 months after the initial surgery, Hartmann reversal was performed, and no evidence of recurrence had been observed at 12 months after surgery.

## DISCUSSION

Approximately 5% of patients with colon cancer present with T4b disease at initial diagnosis, and invasion into adjacent organs such as the small intestine or urinary bladder is frequently evident.^3,5,6)^ Duodenal involvement by colon cancer is uncommon and has been reported predominantly for right-sided tumors, reflecting their anatomical proximity to the duodenum.^2)^ In contrast, the sigmoid colon is a mobile intraperitoneal organ and is usually anatomically distant from the retroperitoneal duodenum; therefore, direct invasion of the duodenum by sigmoid colon cancer is exceedingly rare. A review of the English-language literature identified only 4 previously reported cases of sigmoid colon cancer invading the duodenum, making the present case the fifth to our knowledge. We systematically searched PubMed from inception to January 2026 using the terms “(sigmoid OR sigmoido) AND (duodenal OR duodenum) AND fistula AND (carcinoma OR cancer),” and this identified 3 eligible reports. One additional case report by Minutolo et al., which is not indexed in PubMed, was identified through manual screening of reference lists and citation tracking in Google Scholar, yielding 4 similar cases for detailed comparison.^7–10)^ The characteristics of these 4 cases, along with the present case, are summarized in **Table 1**.

**Table 1:** Summary of reported cases of duodenal invasion by sigmoid colon cancer

Reference	Age/sex	Portion	Procedure for primary tumor	Procedure for duodenum	Surgical intent	Postoperative complications	Postoperative treatment	Outcome
Melissas et al.	76/M	3rd	Sigmoid colectomy	Partial resection (primary closure)	Curative	None	Adjuvant chemotherapy	Alive (2 years) without recurrence
Minutolo et al.	84/M	4th	Sigmoid colectomy	Resection of the 4th portion (duodenojejunostomy)	Curative	None	None	Alive (1 year) without recurrence
Huber et al.	63/F	N/D	Hartmann’s procedure Adnexectomy, ileal resection	Wedge resection (primary suture)	Palliative	None	Local radiotherapy + chemotherapy	N/D
Shapey et al.	91/F	4th	Not performed	―	―	―	―	Died
Present case	66/M	3rd	Hartmann’s procedure	Segmental duodenectomy (duodenojejunostomy + gastrojejunostomy)	Curative	Urinary dysfunction	None	Alive (1 year) without recurrence

F, female; M, male; N/D, not determined

The third portion of the duodenum was involved in 2 cases and the fourth portion in 2, whereas the precise location was not described in 1 case. In all of these reported cases, a preoperative diagnosis was achieved using endoscopy and/or imaging. Similarly, in the present case, a preoperative diagnosis was successfully established using CT and endoscopy. In contrast, the optimal therapeutic strategy remains unclear. Among the 5 reported cases, curative resection including the primary tumor was achieved in only 3 cases. Furthermore, 1 case ultimately required palliative treatment, highlighting the considerable difficulty associated with the surgical management of tumors with duodenal invasion. When tumor invasion extends beyond the duodenum to the pancreas, pancreatic resection including PD becomes necessary, and invasion of major vessels such as the SMA/V further compromises resectability. Subsequently, a considerable number of cases may be deemed unsuitable for surgical intervention due to the magnitude of invasiveness. On the other hand, although extensive duodenal invasion was observed in the present case, no pancreatic involvement was identified; therefore, pancreas-preserving segmental duodenectomy was feasible. Segmental duodenectomy is less invasive than PD and can be performed with a relatively favorable safety profile. Therefore, in cases without pancreatic invasion, such as the present case, this procedure should be actively considered as a surgical option to achieve curative resection. Kato et al. have likewise reported pancreas-sparing distal duodenectomy as a viable alternative to PD for duodenal lesions.^11)^

On the other hand, there is no consensus regarding gastrointestinal reconstruction following segmental duodenectomy, particularly after distal segmental duodenectomy involving the third and fourth portions of the duodenum.^12)^ As a reconstructive option, anastomosis at the duodenal resection margin should be considered the first-line approach if it can be performed safely. However, careful consideration is required in cases where the procedure is technically challenging on the dorsal side of the SMA/V, as well as in situations where there is a risk of potentially fatal leakage at the anastomosis site near the SMA/V, which may result in exposure to pancreatic leakage. For duodenal reconstruction, when utilization of the duodenal stump is not feasible, previous reports have described reconstruction after segmental duodenectomy mainly using duodenojejunostomy alone without additional gastrojejunostomy. Although this approach is technically simple and involves only a single anastomosis, several authors have reported substantial rates of anastomotic fistula formation and DGE after this type of duodenojejunostomy alone.^11,12)^ Kato et al. also reported that DGE occurred in 42% of cases when duodenojejunostomy alone was performed.^11)^ Therefore, in the present case, we created a Roux-en-Y jejunal limb with duodenojejunostomy, followed by additional gastrojejunostomy, with the aim of separating the drainage route of pancreatic juice and bile from the alimentary route.^12,13)^ As a result, DGE did not occur and the patient retained a good appetite.

## CONCLUSIONS

Malignant invasion of the duodenum is often associated with involvement of major vessels or the need for pancreatic resection, which may render curative resection difficult or excessively invasive. However, segmental duodenectomy with preservation of the pancreas can be a valuable surgical option in selected cases. These technical modifications suggest that even in patients with malignant sigmoid–duodenal fistula, a favorable long-term outcome may be achievable if curative resection can be performed successfully.

## DECLARATIONS

### Availability of data and materials

Data sharing is not applicable to this article.
